# Temperature-related body size change of marine benthic macroinvertebrates across the Early Toarcian Anoxic Event

**DOI:** 10.1038/s41598-020-61393-5

**Published:** 2020-03-13

**Authors:** Veronica Piazza, Clemens V. Ullmann, Martin Aberhan

**Affiliations:** 1 Museum für Naturkunde, Leibniz Institute for Evolution and Biodiversity Science, Invalidenstraße 43, 10115 Berlin, Germany; 2 University of Exeter, Camborne School of Mines, College of Engineering, Mathematics and Physical Sciences, Penryn, Cornwall, TR10 9FE UK

**Keywords:** Palaeoecology, Climate sciences, Ecology

## Abstract

The Toarcian Oceanic Anoxic Event (TOAE, Early Jurassic, ~182 Ma ago) was characterised by severe environmental perturbations which led to habitat degradation and extinction of marine species. Warming-induced anoxia is usually identified as main driver, but because marine life was also affected in oxygenated environments the role of raised temperature and its effects on marine life need to be addressed. Body size is a fundamental characteristic of organisms and is expected to decrease as a response to heat stress. We present quantitative size data of bivalves and brachiopods across the TOAE from oxygenated habitats in the Iberian Basin, integrated with geochemical proxy data (δ^13^C and δ^18^O), to investigate the relationship between changes in temperature and body size. We find a strong negative correlation between the mean shell size of bivalve communities and isotope-derived temperature estimates, suggesting heat stress as a main cause of body size reduction. While within-species size changes were minor, we identify changes in the abundance of differently sized species as the dominant mechanism of reduced community shell size during the TOAE. Brachiopods experienced a wholesale turnover across the early warming phase and were replaced by a virtually monotypic assemblage of a smaller-sized, opportunistic species.

## Introduction

Growth, and hence body size of organisms, is affected by both biotic and abiotic factors. Body size decrease in particular is observed during and after episodes of warming in modern and extinct taxa (e.g. refs. ^1–4^). Temperature increase has complex and wide-ranging effects on organisms^5,6^. Once temperature surpasses the optimal thermal range of an organism it can result in decreased growth, reproduction and feeding rates up to the collapse of all biological functions^7^. Body size decrease has thus been suggested as a third universal response to warming^8^ in addition to changes in the geographic distribution of species and in phenological events^1,9^.

We focus on changes in shell size of marine benthic macroinvertebrates across the Early Toarcian Oceanic Anoxic Event (TOAE; Early Jurassic, ~182 Ma)^10^. Only a few studies have addressed this topic, mostly focusing on anoxic settings^11–15^. Marine oxygen depletion has usually been considered the prime causal mechanism of increased early Toarcian extinction rates and faunal turnover^16–18^. Nevertheless, elevated extinction intensities and body size decrease before and across the TOAE have been registered also in well-aerated shallow-shelf settings in the western Tethys (e.g. refs. ^19–22^). This implies that regional environmental differences existed during the TOAE^23^, and that anoxia alone cannot be considered a universal and single driver of the associated biotic crisis. High temperatures during the TOAE have been suggested as an important factor in oxic settings^20,21,24^. However, statistical tests of causal links between fluctuating temperatures with diversity and community composition are exceedingly rare (but see refs. ^25,26^) and so far are lacking regarding the relation between temperature and body size.

We quantitatively evaluate the role of temperature for changes in body size of bivalves and brachiopods from an oxygenated setting in the Iberian Range of Spain. Specifically, we test whether body size is reduced during the hyperthermal conditions of the TOAE, and to which degree body size is correlated negatively with ambient water temperatures. We focus on changes of the average shell size of bivalve-brachiopod communities through time. Essentially, heat stress-induced reductions in mean shell size at the community level can be caused by two mechanism: (1) a decrease in shell size within species as known from the physiological response of modern organisms, and/or (2) a change in species composition, with smaller-sized species replacing or becoming more abundant than larger-sized species.

We obtained body size trajectories by plotting the mean shell size of specimens per faunal sample for bivalve-brachiopod communities as a whole and separately for the bivalve and brachiopod subcommunities and for individual species. Changes in shell size were tested for correlation with the δ^18^O time series, our geochemical proxy for ambient water temperatures, derived from rhynchonellid brachiopods and oyster shells from the same levels as the faunal data. We performed Generalized Least Squares (GLS) fitting on a direct correlation of body size and δ^18^O (here termed lag 0) and on a lagged isotope time series (here termed lag 1), i.e. correlating each body size value with the δ^18^O value of the immediately preceding sample, to test for a delayed response of body size to temperature.

## Studied Section and Environmental Conditions

**Figure 1:**
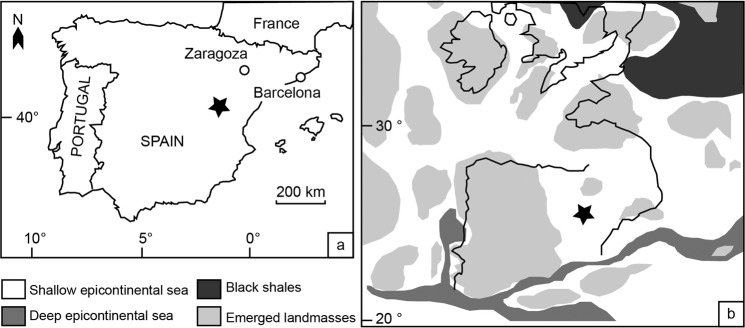
Geographic location of the studied section at Barranco de la Cañada (**a**) and Early Jurassic palaeogeographic reconstruction of the NW Tethys (**b**). Location of the studied area is marked with a black star. Palaeomap modified from Fig. 1 in ref. ^17^ (software used: Adobe Illustrator v. CS6 [https://www.adobe.com/de/products/illustrator.html]).

**Figure 2:**
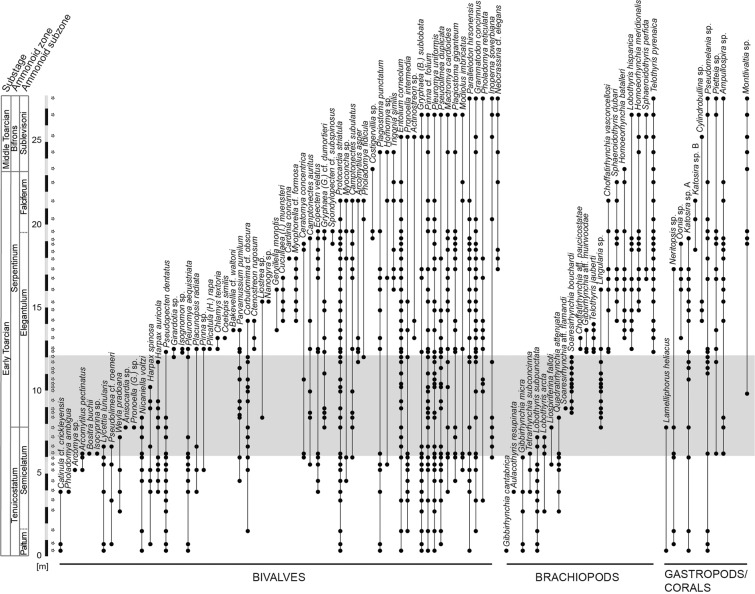
Stratigraphic ranges of benthic macroinvertebrate species at Barranco de la Cañada. The taxa have been ordered by last occurrences, and subdivided into the main taxonomical groups. Each point represents the presence of a taxon in a sampled level. The star-shaped symbols indicate the sampled levels. The chemostratigraphically defined TOAE interval is represented by the shaded grey area. Chronostratigraphic boundaries modified from ref. ^28^.

**Figure 3:**
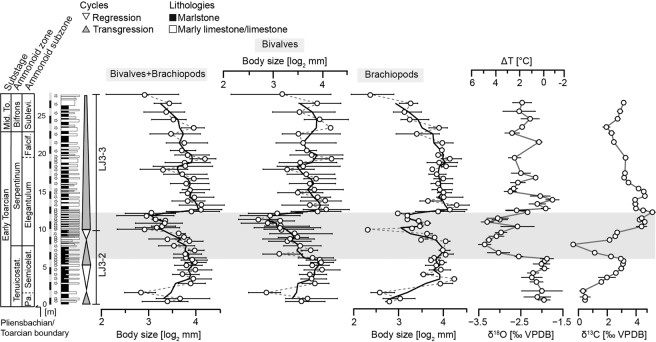
Stratigraphy, mean shell size of all individuals of the three main analysed faunal groupings by sample, and geochemical data (δ^18^O and δ^13^C) of the Barranco de la Cañada succession. The TOAE (grey shading) represents the extent of the globally recognised negative δ^13^C isotopic excursion. Horizontal bars in the shell size time series represent the interquartile range delimited by the first and third quartiles, while in the isotope time series they represent the double standard error for each sample. A three-point moving average (solid black line) was fitted to the faunal data alongside the point-by-point trend (dashed grey line). Isotope data after ref. ^32^ and temperature change ΔT, relative to the lowermost value recorded in the profile, is estimated from the equation in ref. ^35^. Third-order transgressive-regressive cycles from refs. ^29,30^. Abbreviations: Falcif. = Falciferum; Mid. To. = Middle Toarcian; Pa. = Paltum; Semicelat. = Semicelatum; Sublevi. = Sublevisoni; Tenuicostat. = Tenuicostatum.

We studied a ca. 28-m-thick sedimentary succession at Barranco de la Cañada (40°23′53.4″N 1°30′07.4″W) near Albarracín, Spain (Fig. 1), where the Toarcian Turmiel Formation^27^ is characterised by rhythmic alternations of marlstones and partly argillaceous limestones. The latter primarily comprise mudstones, wackestones, and floatstones indicative of low-energy conditions below storm wave base at an estimated water depth of 40–70 m^28–30^. Packstones and rudstones are very rare, suggesting transient episodes of higher water energy, interpreted as distal storm flow beds^19^. Deposition took place in a well-oxygenated mid-ramp setting. Black shales typical for the TOAE elsewhere are absent. The section has a well-defined biozonation based on ammonites and brachiopods^31^, and is highly fossiliferous, with abundant benthic macroinvertebrates (Fig. 2). The studied interval ranges from the Pliensbachian/Toarcian boundary to the lower Bifrons Zone of the middle Toarcian. We identified the TOAE chemostratigraphically using the characteristic negative carbon-isotope excursion (CIE)^16^ obtained from well-preserved shells of rhynchonellid brachiopods and oysters (Fig. 3). The TOAE thus spans the uppermost Tenuicostatum Zone up to the lower Serpentinum Zone.

Oxygen isotope ratios from the same specimens provide a record of palaeotemperatures at the seafloor. Across the studied interval the measured isotope ratios indicate a rapid increase in temperatures when the negative CIE of the TOAE commenced. Palaeotemperatures then stayed stable and high until the end of the negative CIE, where a brief return to pre-excursion temperatures is indicated. The upper part of the section is then characterised by a slight warming, resulting in average water temperatures intermediate between earliest Toarcian temperatures and those during the negative CIE. Temperature estimates indicate that palaeotemperatures were elevated by ~3.5 °C throughout the TOAE^32^ (Fig. 3; see Methods and Fig. S1 for details).

In terms of sequence stratigraphy (summarized in refs. ^28–30,33^) the studied interval is part of a second-order transgressive-regressive cycle (LJ-3 in ref. ^30^) ranging from the early Pliensbachian (Davoei Zone) to the middle Toarcian (Variabilis Zone). The transgressive phase occurred in three distinct pulses, of which the second (LJ3-2) and parts of the third (LJ3-3) third order transgressive-regressive cycle fall within the study interval^30^ (Fig. 3).

## Results

### Changes in shell size

A temporary decrease in mean shell size during the TOAE is clearly evident in the high-resolution trajectories of the bivalve-brachiopod communities and subcommunities (Fig. 3). They illustrate that the brachiopod and bivalve subcommunities start becoming smaller-sized at different times. Shell size of bivalve subcommunities starts to decrease at the beginning of the TOAE simultaneously with the first shift to warmer temperatures, whereas brachiopod subcommunities only get smaller in the later part of the TOAE. The re-establishment of pre-TOAE shell sizes of all community types occurred synchronously and rapidly at the termination of the TOAE (Fig. 3). This rapid recovery of mean body size corresponds to a distinct shift to cooler temperatures, suggesting an important role of warming in controlling reductions in shell size and of cooling in the subsequent return to original size distributions.

**Figure 4:**
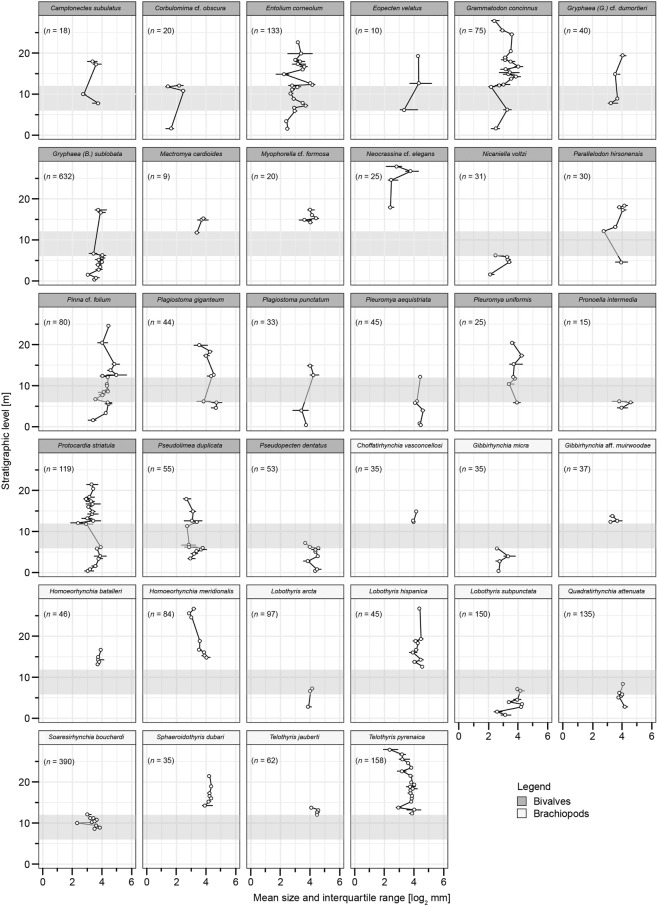
Mean shell size of all individuals by sample of bivalve and brachiopod species present in at least three levels and with at least three specimens per sample. Interquartile ranges represented by the black horizontal lines. TOAE represented by the grey shading. *n* = total number of individuals.

Within-species size patterns of the more common species, i.e. those 34 species that occur with at least three specimens per level and at least at three sampling levels, are different for bivalves and brachiopods (Fig. 4). Most of the common brachiopod species are confined to either the pre-TOAE, the TOAE, or the post-TOAE interval (Figs. 2 and 4). Only three common species extend from pre-TOAE times into the lower part of the TOAE before they disappear without appreciable change in shell size (Fig. 4). In the upper part of the TOAE they are replaced by the relatively small-sized brachiopod *Soaresirhynchia bouchardi*. Bivalve species, by contrast, commonly occur in two or all three intervals (Figs. 2 and 4). Unequivocal indication for consistently reduced size of individual bivalve species during the TOAE hyperthermal is missing. Yet, several species show a moderate size decrease at the beginning of the TOAE relative to the preceding sampling levels (*Gryphaea (B.) sublobata*, *Nicaniella voltzi*, *Pinna* cf. *folium*, *Plagiostoma giganteum*, *Pronoella intermedia*, *Pseudolimea duplicata*, *Pseudopecten dentatus*) or a size increase from the end of the TOAE into the early post-TOAE phase (*Entolium corneolum*, *Grammatodon concinnus*, *Parallelodon hirsonensis*, *Pseudolimea duplicata*).

### Abundance change of larger-sized *versus* smaller-sized species

**Figure 5:**
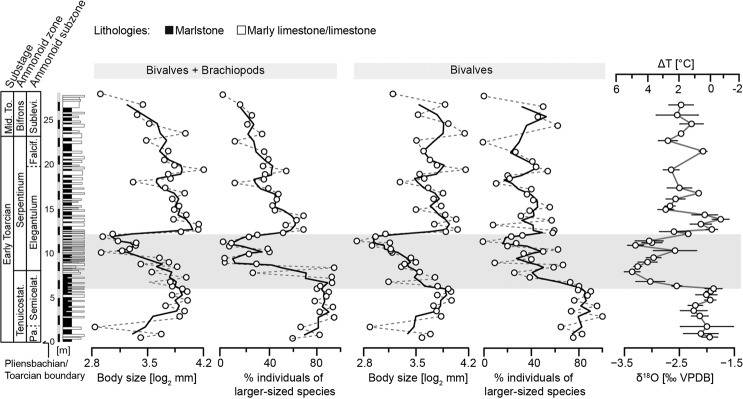
Relative abundance, expressed in percentage, of all individuals of larger-sized species in each sample for the whole bivalve–brachiopod community and for bivalves. The two time series are plotted alongside the mean shell size of the corresponding group and the δ^18^O isotope time series from Fig. 3. For further explanations, see Fig. 3.

To assess the impact of changes in the relative abundance of differently sized species for size change at the community scale, we grouped the common species into “smaller-sized” and “larger-sized” using the mean of all species’ mean shell sizes as the cut-off value (see Methods, Supplementary Fig. S2). Of the 34 common species, 19 are categorized as larger-sized (11 bivalves, 8 brachiopods) and 15 as smaller-sized (10 bivalves, 5 brachiopods). To facilitate comparison we plotted the percentage of individuals of larger-sized species per sample next to the already established trajectories of mean community shell size and of inferred bottom water temperature change (Fig. 5 and Supplementary Fig. S3). For bivalve-brachiopod communities, overall trends in mean shell size and in abundance of larger-sized species are strikingly similar and significantly correlated (Table 1). Clearly, the transient decrease in mean community level shell size during the TOAE is associated with a decline in the relative abundance of larger-sized species. Similar patterns are evident in bivalve and brachiopod subcommunities (Table 1), where a decline in the abundance of larger-sized species during the TOAE is reflected by smaller community shell sizes than during pre-TOAE times (Fig. 5 and Supplementary Fig. S3).

**Table 1:** Results of the Generalized Least Squares (GLS) correlation between shell size and relative abundance of individuals of larger-sized species for the three analyzed groups (brachiopod + bivalve community; brachiopod and bivalve subcommunities).

Analyzed group	ARIMA(p,d,q)	*p*-value	Correlation	Residual standard error
Brachiopods + Bivalves	(0,0,0)	**<0.001**	−0.039	16.735
Brachiopods	(0,0,0)	**0.001**	−0.048	22.618
Bivalves	(0,0,0)	**<0.001**	−0.033	17.404

Time series were differenced before correlation because of the presence of trends in time and autocorrelation. Abbreviations of the parameters of the AutoRegressive Integrated Moving Average (ARIMA) fitted to the GLS model: p = number of time lags; d = degree of differencing; q = order of moving average. Statistically significant values are in bold.

### Correlation of shell size and species abundance with temperature

Tests for autocorrelation and stationarity of the faunal and isotope data (see Methods and Supplementary Tables S1–S4) did not flag any caveats for the applicability of GLS regression on the original body size time series, while differencing was necessary when the relative abundance of larger-sized species was part of the model. The correlation of δ^18^O values and shell size of bivalve-brachiopod communities is significant at lag 1, suggesting that the temperature at one sample level was influencing shell size in the next younger sample (Table 2). Correlation at lag 0, by contrast, is not significant. For the bivalve subcommunities, body size is significantly correlated with δ^18^O for both lag 0 and lag 1, while in brachiopods the correlation is non-significant in both cases (Table 2). These results, along with differences in the timing of size changes, hint at a decoupled response to changes in water temperature of these two groups. Regarding the relative abundances of larger-sized species, results for bivalve-brachiopod communities and bivalve subcommunities show significant correlations with δ^18^O at lag 0 (Table 2).

**Table 2:** Results of the Generalized Least Squares (GLS) correlation between shell size/ percentage of larger-sized individuals and δ^18^O for the analyzed faunal groups.

Analyzed group	ARIMA(p,d,q)	*p*-value	Correlation	Residual standard error
**Body size (Brachiopods + Bivalves)**				
Lag 0	(0,0,0)	0.123	0.984	0.297
Lag 1	(0,0,0)	**0.050**	0.984	0.291
**Body size (Brachiopods)**				
Lag 0	(1,0,0)	0.327	0.961	0.430
Lag 1	(0,0,1)	0.380	0.976	0.391
**Body size (Bivalves)**				
Lag 0	(0,0,0)	**<0.001**	0.984	0.313
Lag 1	(0,0,0)	**0.007**	0.984	0.337
**% Large individuals (Brachiopods + Bivalves)**				
Lag 0	(0,0,0)	**0.023**	−0.012	21.202
Lag 1	(0,0,0)	0.833	−0.016	21.549
**% Large individuals (Brachiopods)**				
Lag 0	(0,0,0)	0.495	−0.008	25.828
Lag 1	(0,0,0)	0.124	−0.013	22.704
**% Large individuals (Bivalves)**				
Lag 0	(0,0,0)	**0.015**	−0.011	19.460
Lag 1	(0,0,0)	0.501	−0.017	22.755

Lag 0: Correlation of body size values with δ^18^O values for the same sample. Lag 1: Correlation of body size values with the δ^18^O values of the immediately preceding sample. For specifications of the parameters of the AutoRegressive Integrated Moving Average (ARIMA) see caption of Table 1. Statistically significant values are in bold.

## Discussion

### Excluding biases in size frequency distributions

Habitat change can be a potential source of bias when investigating body size trends, as variations in body size can originate from facies changes due to fluctuations in relative sea level. If body size were primarily controlled by changes in sea level, we would expect similar fluctuations in size in each transgressive-regressive cycle. Because this is not the case (Fig. 3), and the represented depositional environments stayed below or close to storm wave base throughout the studied time interval, we conclude that fluctuations in sea level and depositional environments did not generally drive the observed variations in shell size. Furthermore, lack of size sorting and the absence of preferential orientations of shells suggest that post-mortal shell transport was minor and that the frequency distributions of shell size were not modified by currents or waves. We infer that the observed size patterns were not driven by changes in relative sea level and depositional environments, and were not biased by taphonomic processes, but rather demonstrate a negative effect of rising temperatures on shell size of benthic macroinvertebrate assemblages.

### Alternative factors causing the size changes

Apart from heat stress, the observed reductions in community-level shell size could also be caused by other stressors – in particular reduced oxygen concentrations, ocean acidification, low food supply, and seawater freshening – and these factors could have acted in synergy.

#### Salinity

Potential changes in salinity are relevant not only because they could provoke size decrease^34^ but because they would affect the oxygen isotope composition of seawater and thereby affect our temperature estimates. Palaeotemperatures from δ^18^O values in fossil calcite are calculated using equations that require an estimate of the isotopic composition of ambient seawater. Whilst the oxygen isotope thermometer of ref. ^35^ is linear and therefore insensitive to the exact δ^18^O value of the local seawater, biased temperature information may nevertheless derive from substantial salinity changes across the studied time interval.

The magnitude of the oxygen isotope shift from the earliest Toarcian to the TOAE is 1.00 ± 0.09‰ for the studied site. To explain such a shift purely by salinity change, strong freshening of the local water mass would have had to occur. Comparison of the Spanish isotope record to multiple correlative records from other European basins suggests that large scale freshening of waters for which there is some evidence in central and northern Europe (e.g., ref. ^36^) is much more subdued in Spain, if at all present^37,38^. In a shallow, but open marine shelf setting facing deep marine Tethyan basins to the South East (e.g., ref. ^39^), sustained salinity reduction is much less likely to have occurred than in the more restricted subbasins of the Laurasian seaway further north.

At palaeolatitudes of ~25 °N, as reconstructed for the studied basin (Fig. 1; ref. ^39^), annual average meteoric water signatures are very unlikely to have been < −5‰^40^. Salinity-induced reduction of ambient water δ^18^O values is therefore expected to be 0.1‰ per psu or less. Lethally low salinity levels for the consistently observed stenohaline fauna (ammonites, crinoids, and rhynchonelliform brachiopods are continuously present throughout the section and specifically in the TOAE interval) would have occurred already much before ambient water δ^18^O would have changed by 1‰. We therefore conclude that large salinity changes are not compatible with faunal assemblages, considerations of meteoric signals, and comparison to other European basins. Consequently, we interpret the vast majority of the measured oxygen isotope signals during the TOAE to be a consequence of temperature change.

#### Hypoxia

Regarding the oxygen concentrations of bottom waters there is no evidence of severe hypoxia in the benthic environments of Barranco de la Cañada. Black shales, commonly characterising the TOAE elsewhere, are absent, shelly macroinvertebrates are ubiquitous, and bioturbation is common with a diverse suite of ichnotaxa (*Spongeliomorpha*, *Thalassinoides*, *Palaeophycus*, *Rhizocorallium*, *Sphaerichnus*, *Trichichnus*) in the TOAE interval (ref. ^28^; own observations). The ichnogenera *Spongeliomorpha* and *Thalassinoides* are produced by crustaceans, and because this faunal group is sensitive to hypoxia^41^, their appearance is independent evidence against low-oxygen conditions on and within the seafloor^22^.

#### Other environmental factors

As a direct consequence of elevated levels of CO_2_ in the atmosphere, global warming is commonly associated with ocean acidification. From experiments with modern species it appears that bivalves are more negatively affected by ocean acidification than articulate brachiopods^22,42,43^. This pattern of inferred physiological pH tolerance of bivalves and brachiopods does not conform with the Early Toarcian faunal pattern at Barranco de la Cañada, where most brachiopod species went extinct during the TOAE^24^ (see Discussion below). Thus, acidification cannot be inferred from the faunal patterns at Barranco de la Cañada, but independent signs of ocean acidification, such as changes in the boron isotope composition, should be investigated. Finally, a decline in primary productivity could be a stressor affecting specifically species with high energy demands such as active and/or large-sized taxa. The selective extinction of brachiopods argues against a low-productivity scenario because their low metabolic rates^44^ and their capability to feed on particulate and dissolved organic matter^45^ should buffer them against reduced food supply (but see ref. ^19^).

### Temperature-related mechanisms of size change

As temperatures rise, organisms tend to get smaller, a pattern broadly known as the temperature–size rule that is widespread in marine ectotherms^46^. In general, organisms tend to have higher metabolic rates, faster growth, and mature earlier in the tropics than those in polar regions, which in turn have longer lifespans often leading to larger final size^47,48^. Modern marine bivalves also show the common pattern of increasing lifespan and decreasing growth rates with latitude (a proxy for temperature)^49^, but at large do not follow Bergmann’s rule (the assertion that body size increases with latitude)^50^. This lack of a broadly generalizable latitudinal size gradient within and across bivalve taxa is explained by opposing trends in lifespan and growth rate with latitude that cancel each other out^49^. Still, we can expect within-species size reductions during episodes of warming at any given latitude if populations shift toward the upper thermal limits of their thermal niche. Of central importance for the performance of organisms with temperatures rising beyond their thermal optimum is that they maintain the scope for aerobic activity in spite of increasing oxygen demand^6,51^. Once the mismatch between oxygen supply capacity and oxygen demand exceeds a critical threshold, physiological functions are affected negatively, leading, amongst other effects, to reduced growth and finally mortality^52^.

Our results, however, suggest only minor size reductions within some bivalve species limited to the beginning and the end of the hot TOAE interval (Fig. 4). These somewhat lowered size values might have a small cumulative effect in reducing community level shell size during the TOAE but cannot explain the magnitude of the actual size reductions. Rather, many bivalve species present before and after the TOAE were rare during the TOAE, and changes in species composition are even stronger in brachiopods (Figs. 2 and 4). Apparently, the local populations were not generally able to adapt to the persistently warmer temperatures and any related changes that existed during the TOAE interval.

Thus, the primary mechanism of size change in the studied bivalve and brachiopod communities are variations in species composition (Figs. 2 and 4) and in the relative abundance of differently sized species, with smaller species prevailing during the TOAE (Fig. 5, Supplementary Fig. S3). Specifically, the size decline during the TOAE is paralleled by relative abundance declines in larger-sized species, and both ecological variables are significantly correlated with bottom water temperatures as deduced from the oxygen isotope record (Table 2). Changes in community composition and species abundance are not only compatible with the observed lag 0 correlations but also with the lag 1 correlations that indicate an effect with a brief temporal offset between changes in water temperature and subsequent changes in community shell size. Greater susceptibility of larger *versus* smaller aquatic ectotherms is known from modern marine ecosystems. Locally, loss of larger specimens of a species occurs first, followed by enhanced mortality, decline in reproduction rates, and local extinctions^53,54^. Meta-analyses of extant aquatic ectotherms showed a significant increase in the proportion of small-sized species in warming oceans^1^, and a more negative temperature–size relationship in larger than smaller species^47^. Again, higher oxygen requirements in larger organisms have been proposed to explain this difference in short-term responses to warming^47,53^, but other factors such as energy balance may have become important in the long-term^54^.

### Variable response of bivalves and brachiopods to warming

The size response of bivalves and brachiopods to temperature change appears to be decoupled at the onset of the warming phase (Fig. 3). Bivalves show a strong temperature-size correlation (Table 2). They did not experience unusually intense extirpations (Fig. 2) but warming affected larger-sized species more negatively than smaller-sized species. Physiological experiments have shown that molluscs are amongst the most tolerant invertebrate groups towards increased temperatures (summarized in ref. ^5^), presumably because they tend to be more active organisms that have a higher aerobic scope to meet increased oxygen demands at raised temperatures^54,55^ than, for example, sessile brachiopods. This is consistent with our data: despite size-specific changes in species composition and abundance during the warm TOAE interval, most bivalve species ranged through the TOAE (Fig. 2).

Conversely, the brachiopods thriving before the TOAE disappeared completely during the early warming phase (Fig. 2). None of the pre-TOAE articulate brachiopod species of the Iberian platform system survived this episode^24^. In contrast to bivalves, shell size of brachiopod-subcommunities remained stable during the distinct temperature rise in the early phase of the TOAE (Fig. 3), a pattern also reported from other parts of the Iberian Range^24^. The shift to smaller sizes in the upper part of the TOAE hyperthermal is entirely due to the appearance of a smaller-sized brachiopod species, the basiliolid *Soaresirhynchia bouchardi*, at the concurrent absence of larger-sized brachiopod species (Figs. 2 and 4 and Supplementary Fig. S3).

Information on the thermal tolerance of brachiopods is scarce, and it is thus difficult to predict their response to warming. Available evidence from the Antarctic rhynchonellid *Liothyrella uva* points at lower thermal tolerance of this species compared to bivalves from the same environment^56^. Despite the absence of a significant correlation between shell size and temperature for brachiopods across the whole studied time interval (Table 2), brachiopod turnover and extinctions in the early part of the TOAE hyperthermal suggest that they reacted more sensitively to heat stress than bivalves, conforming to the observations of ref. ^56^. Furthermore, the high abundances of the relatively small-sized *Soaresirhynchia bouchardi* in the later part of the TOAE are noteworthy (Fig. 4). It is regarded as a slow-growing species of deeper water origin belonging to a group that migrated into shallow shelf settings during the environmental perturbations of the TOAE, and that succeeded by opportunistically exploiting the ecological space left unoccupied after the extinction of the pre-existing brachiopod fauna^32,57^. Reduced size suggests reduced metabolic rates, which makes organisms like *S. bouchardi* more tolerant against increasing temperatures (and other environmental stressors) than larger-sized organisms with higher metabolic demands^58^. We suggest that also the shift in brachiopod composition in the TOAE interval towards populations of relatively small-sized *S. bouchardi* was a consequence of concomitant temperature stress. With regard to anthropogenic climate warming and its ecological effects on marine life in the near future, a similar proliferation of relatively small-sized immigrants and long-term reductions in community size structure are worrying perspectives.

## Conclusions

We find strong evidence that shell size of macrobenthic communities at Barranco de la Cañada was overall smaller during the environmental crisis represented by the TOAE than before and afterwards. Rather than size change within individual species the key mechanism of size reduction was a decline in the presence and abundance of larger-sized species.

While low oxygen conditions can be excluded as a main driver of shell size reductions in the well oxygenated environments studied herein, we show that temperature correlates negatively particularly with the average shell size of bivalve subcommunities and with the percentage of larger-sized bivalves. Also, for brachiopods, albeit not supported statistically in the temperature–size time series correlation, we argue that extinction-related turnover and the success of a smaller-sized disaster species during the crisis are best explained by seawater warming. Obviously, the nature of environmental stresses varied geographically and it remains unclear at present to which degree additional drivers such as ocean acidification, and altered nutrient regimes may have influenced the observed faunal patterns. We conclude that the long-standing paradigm of severe oxygen restrictions as the main cause of faunal change during the TOAE needs to be modified by including heat stress as an important environmental factor.

## Methods

### Sampled material and databases

We performed quantitative bed-by-bed sampling from carbonate beds and we standardized sampling intensity by consistently collecting ~13 kg of bulk rock for each sample. Sampling focused on the complete coverage of shelly benthic macroinvertebrates, regardless of preservation quality and without preferential extraction of smaller or larger specimens. Specimens were identified at species level as far as preservation quality allowed.

We recorded dorsal valves, ventral valves, and double-valved specimens for brachiopods and left, right, and articulated valves for bivalves. Measurements were inferred when a small fraction of the shell was missing, while unidentifiable and strongly fragmented specimens were disregarded. Each specimen was considered as one individual, after confirming that the shell sizes of right and left valves of the same species in any sample were different and therefore did not stem from the same individual. Sampling yielded a total of 3,668 occurrences of 93 taxa (62 bivalves, 21 brachiopods, 9 gastropods and 1 coral) (Fig. 2). Because gastropods and corals are rare and fragmented we excluded them from all quantitative analyses.

We performed our analyses on two types of data, quantitative faunal data (shell size) and geochemical data (oxygen isotopes). The shell size dataset is composed of the measurements taken in the field and/or after preparation with digital calipers to the nearest 0.1 mm, accounting for 2,408 measured specimens of bivalves and brachiopods. To estimate the size of communities as a whole we assigned a size value also to specimens for which no direct size measurements were available. These size values were calculated as the mean size of the species in the respective sample. If no measurements were available for a species in a sample we used the mean size of that species in the samples directly above and below. The few taxa with insufficient size data were deleted. The final dataset is composed of 3,433 specimens of bivalves and brachiopods, of which 30% of the measurements were inferred indirectly. To evaluate whether this procedure influenced our analytical outcome we compared our results with those obtained from using just the actually measured specimens. Both analyses yielded congruent size patterns and statistical results. Specimens collected in the field are deposited at the Museo de Ciencias Naturales de Universidad de Zaragoza (ref. ^59^; inventory numbers MPZ 2019/415 – MPZ 2019/571, MPZ 2019/792 – MPZ 2019/889, MPZ 2019/920 – MPZ 2019/942 and MPZ 2019/1041).

### Isotope data

Detailed description of sampling routines, and assessment of fossil preservation and validity of geochemical signatures for palaeoenvironmental interpretation are presented in ref. ^32^. In brief, fossil shell material was extracted after removal of any altered crusts and sediment using a preparation needle or scalpel. For brachiopods fibrous, secondary shell layer material was outlined with the preparation needle and levered off the shell with the needle checking for any possible contamination with altered material or bulk rock matrix. Only visually clean and well-preserved material was accepted for further analysis. *Gryphaea* material was treated in the same way, but where the shell was compact and too hard for efficient use of a preparation needle, a scalpel was employed to shave off shell calcite from the fossil surface. Typically, a few milligrams of calcite were stored in individual containers for further geochemical analysis. Time averaging as a function of sample size and ontogeny is known to affect the amplitude of sclerochronological proxy signals^60^. In order to minimize biases resulting from widely different shell quantities, a target sample size of 4 mg was chosen, which allowed to replicate measurements in case one or several analyses would have failed. Oyster shell increments were typically extracted from comparatively wide areas crossing as few as possible growth increments. For brachiopods shell material was taken parallel to the orientation of shell fibres, and therefore somewhat oblique to growth direction, which may have led to some dampening of seasonal signals extracted from these shells. The observed isotopic variability within individual fossils of typically about 1 permil suggests that resolution of the data is generally significantly higher than yearly. However, in the absence of clear morphological markers of growth rate, the exact time increments covered by single samples cannot be estimated precisely.

Approximately 500 µg of calcite were taken for C and O isotope analysis using the Sercon 20–22 gas source isotope ratio mass spectrometer at the University of Exeter’s Penryn campus employing the continuous flow technique. The calcite material was put into borosilicate glass containers, sealed with rubber septa, and flushed with He for 80 s to remove atmospheric contaminants. After subsequent manual injection of c 100 µL of nominally anhydrous H_3_PO_4_ and reaction at 70 °C, the samples were measured together with two in-house isotope standards (CAR, Carrara Marble; NCA, Namibian Carbonatite), allowing for correction for instrumental drift and biases. Reproducibility of the measurements was controlled by the results of CAR and was found to be ± 0.07‰ (2 sd) for δ^13^C and ± 0.15‰ (2 sd) for δ^18^O, both for the standard analyses in conjunction with studied samples (n = 125) and for the entire year 2018 (n = 917).

To control ultrastructure preservation, a fragment of shell from each studied fossil specimen was also submitted to scanning electron microscope analysis using a FEI Quanta 650 Field Emission Gun SEM at the University of Exeter’s Penryn Campus. For further quantification of shell preservation, about 500 µg of calcite per sample were analysed for element/Ca ratios using an Agilent 5110 VDV ICP-OES at the University of Exeter’s Penryn Campus. Ultrastructure preservation of the shell material was found to be mostly very good to excellent, with brachiopod samples showing only minor features of surficial shell dissolution but lack of neomorphic calcite and recrystallization. Samples exceeding Mn/Ca ratios of 0.1 mmol/mol and/or Fe/Ca ratios of 1 mmol/mol were rejected from further interpretation. Isotopic signals were tested for taxonomic effects and potential metabolic effects to ensure that compositional changes of fossil assemblages did not lead to biased interpretation. Both oysters and secondary shell layer calcite of rhynchonellid brachiopods are thought to represent excellent substrates for palaeotemperature extraction for the Mesozoic essentially devoid of vital effects^61,62^. Differences between brachiopods and *Gryphaea* as well as individuals of different brachiopod taxa in our dataset were undistinguishable from differences between individuals of a single taxon, leading us to conclude that inter-species bias and vital effects can be ignored for the present dataset.

Carbon isotope ratios of the studied specimens were used solely to constrain the extent of the well-known, large, Early Toarcian negative carbon isotope excursion (e.g., ref. ^63^), confirming ammonite biostratigraphy and increasing stratigraphic resolution of the dataset.

Palaeotemperatures were derived from oxygen isotope ratios of samples that passed screening for alteration and were computed using the brachiopod-specific oxygen thermometer of ref. ^35^:

$${\rm{T}}\,^\circ {\rm{C}}=16.192-3.468\ast ({\delta }^{18}{{\rm{O}}}_{{\rm{calcite}}}-{\delta }^{18}{{\rm{O}}}_{{\rm{seawater}}}-{{\rm{Mg}}}_{{\rm{calcite}}})$$

using an average MgCO_3_ content of 0.4 wt% of the fossil shell calcite which has minimal effects on the resulting temperature. Like all other conventional oxygen isotope thermometers this equation requires an estimate of ambient water composition which we set at −1‰ V-SMOW in line with other work published for the studied area and time interval (e.g., ref. ^64^). For the derivation of relative temperature change as pictured in the present study the ambient water composition, however, is irrelevant as long as it did not change substantially across the studied interval, because the sensitivity of the oxygen thermometer of ref. ^35^ is constant. Salinity changes as major driver of the isotopic signal were discounted (see Discussion).

### Statistical methods

Shell size is calculated as the log_2_ of the geometric mean of two dimensions. The geometric mean was determined using the following formulae^65^, where L = shell length, W = shell width and H = shell height:

$${{\rm{GeoMean}}}_{{\rm{Brachiopods}}}=\sqrt{{\rm{L}}\ast {\rm{W}}}\,{\rm{and}}\,{{\rm{GeoMean}}}_{{\rm{Bivalves}}}=\sqrt{{\rm{L}}\ast {\rm{H}}}$$

Shell size analyses for the brachiopod subcommunities were performed on articulate brachiopods only.

Calculating the percentage of larger-sized individuals per sample is based on the common species only, i.e. those that occur with at least three specimens per level and at at least three sampling levels. We calculated the mean shell size of each of these common species and species where classified as either smaller- or larger-sized by using as a cut-off the global mean of the individual species’ mean shell size (Supplementary Fig. S2). The relative abundance of individuals of larger-sized species per sample, expressed as percentage relative to the total count of individuals of common species in each sample, was calculated and the trajectory fitted with a three-point moving average.

#### Correlation tests

Before determining the degree of correlation between δ^18^O values with shell size and with the percentage of larger-sized individuals through time, we deleted the rows with missing values in any of the two time series.

The following steps were performed before the Generalized Least Square (GLS) regression (gls function in “nlme” package^66^), the results of which are summarized in Supplementary Tables S1–S4: (i) the presence of any trend in time was investigated for each time series (shell size/percentage of larger-sized individuals ~ sampling level, and δ^18^O ~ sampling level) through a simple Ordinary Least Squares (OLS) regression and with a Spearman’s Rank correlation test for a comparison; (ii) we checked for autocorrelation and partial autocorrelation of the relationships shell size/percentage of larger-sized individuals ~ δ^18^O through the acf function in base R and durbinWatsonTest function from the “car” package^67^. The original time series were kept for further analyses in cases of non-significant *p*-values from step (i) and/or no serious autocorrelation issues from step (ii); otherwise the time series was detrended via generalized differencing^68^ with the R script available at ‘http://www.graemetlloyd.com/pubdata/functions_2.r’; (iii) we fitted an ARIMA process with the auto.arima function in the “forecast” R package^69,70^ to the residuals from OLS regression of shell size/percentage of larger-sized individuals ~ δ^18^O. Because it is possible that unit roots are present as a different facet of an ARIMA process, the residual series of the fitted ARIMA process was investigated with the ndiffs function from the “forecast” package (with kpss and adf stationarity tests and maximum fifth order differencing applied). Given the negative outcomes of the tests (i.e. no differencing required), we proceeded with the GLS proper; (iv) the ARIMA fit was finally applied in the GLS regression to specify the expected correlation structure of the residuals. All analyses were performed in R (v. 3.5.3^71^).

## Supplementary information

Supplementary information

Supplementary information 2.

## Data Availability

The datasets generated and analysed during the current study are available in the Dryad Digital Repository at 10.5061/dryad.rr4xgxd5v.
