# Supplementary material for: Temperature-related body size change of marine benthic macroinvertebrates across the Early Toarcian Anoxic Event
Source: Sci Rep. 2020 Mar 13;10:4675. doi: 10.1038/s41598-020-61393-5 (PMC7069967; doi:10.1038/s41598-020-61393-5)
Supplement: Supplementary file 1 — Supplementary information [file 41598_2020_61393_MOESM1_ESM.pdf]

Supplementary Material for

“Temperature-related body size change of marine benthic macroinvertebrates across the Early Toarcian Anoxic Event”

Veronica Piazza[1 (*)], Clemens V. Ullmann[2], and Martin Aberhan[1]

[1]*Museum für Naturkunde, Leibniz Institute for Evolution and Biodiversity Science, Invalidenstraße 43, 10115 Berlin, Germany. E-mail: *Veronica.Piazza@mfn.berlin; Martin.Aberhan@mfn.berlin*

[2]*University of Exeter, Camborne School of Mines, College of Engineering, Mathematics and Physical Sciences, Penryn, Cornwall TR10 9FE, UK. E-mail: C.Ullmann@exeter.ac.uk*

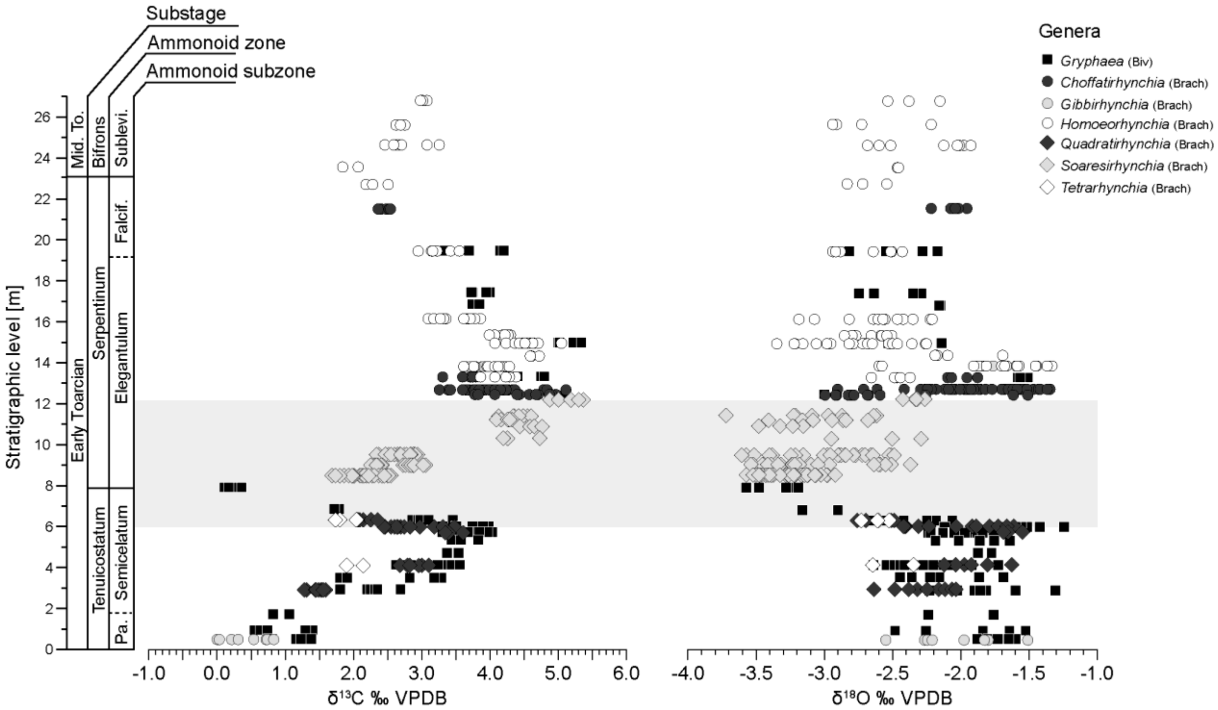

Figure S1. Carbon and oxygen isotope ratios for all individual samples from the Barranco de la Cañada section that passed screening for diagenesis. The data points are coded to indicate the genera that were sampled for isotope analyses at each sampling level. Abbreviations: "Biv" = Bivalve, "Brach" = Brachiopod. For further information, see caption of Supplementary Fig. S3.

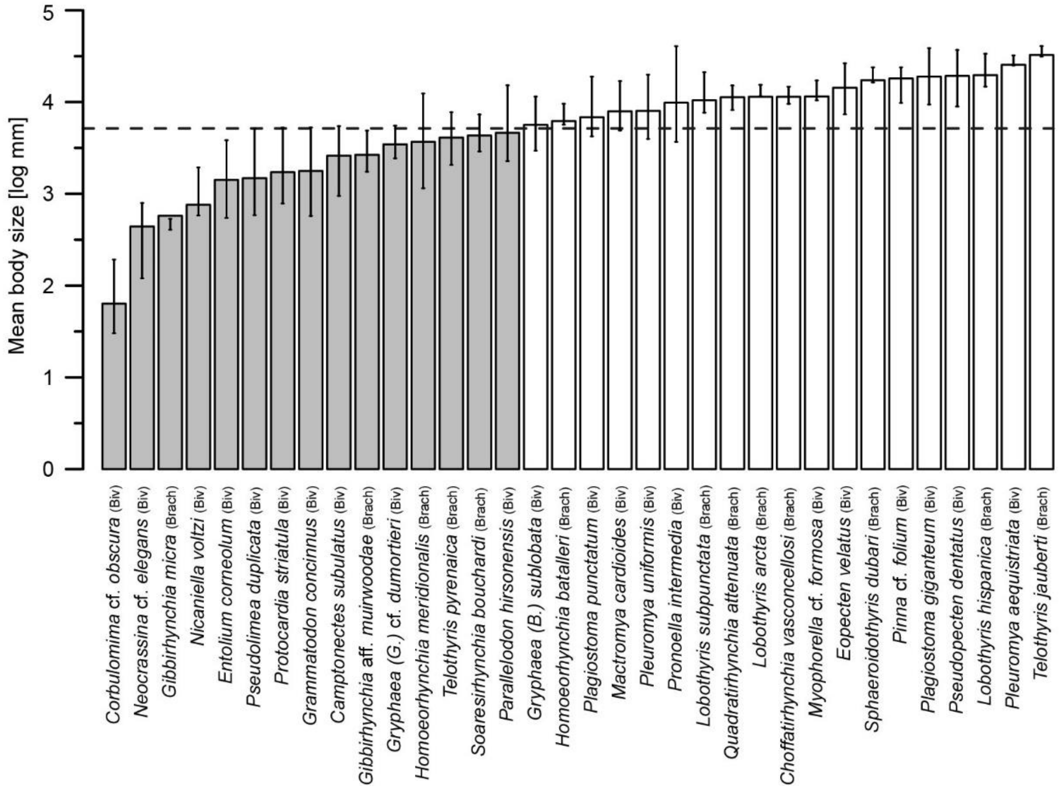

Figure S2. Mean within-species shell size of common species that occur at at least three levels and with at least three specimens per sample at Barranco de la Cañada. The vertical lines denote interquartile size ranges. The horizontal dashed line represents the global mean of the individual species' mean shell size. Species are accordingly classified as smaller- (grey bars) and larger-sized (white bars). Abbreviations: "Biv" = Bivalve, "Brach" = Brachiopod.

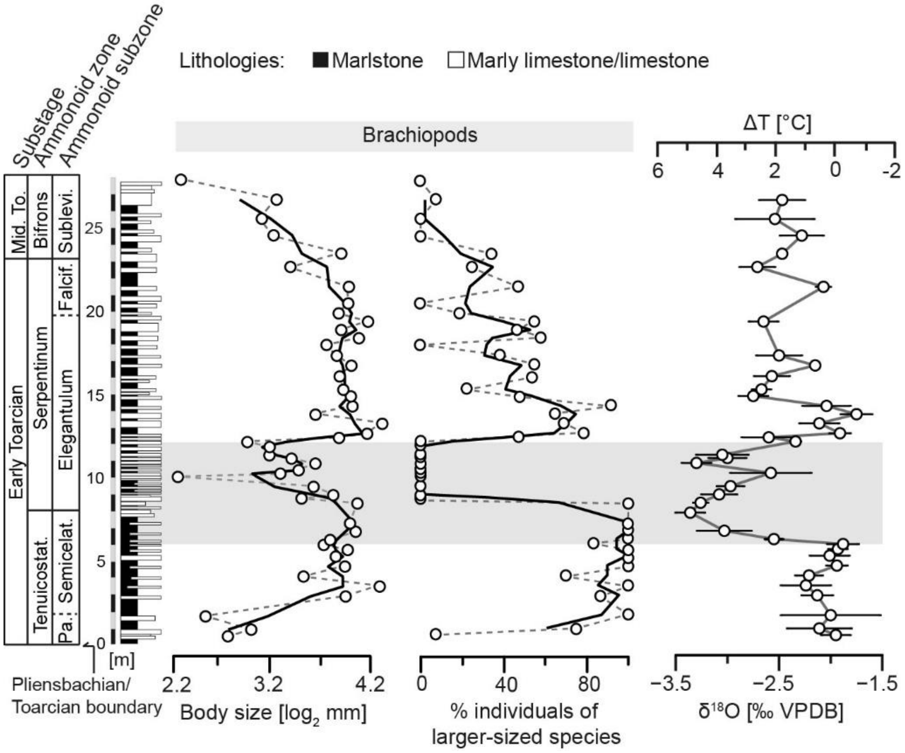

Figure S3. Relative abundance, expressed as percentage, of larger-sized species by sample for the brachiopod subcommunity, plotted next to the mean shell size of the brachiopod subcommunity and $\delta^{18}O$ isotope values (both from Fig. 3). The temperature change ΔT is relative to the lowermost value recorded in the profile and is calculated with the equation from ref.[1]. The grey shading represents the TOAE. Abbreviations: Falcif. = Falciferum; Mid. To. = Middle Toarcian; Pa. = Paltum; Semicelat. = Semicelatum; Sublevi. = Sublevisoni; Tenuicostat. = Tenuicostatum.

Table S1

	**Shell Size ~ Level**				**% Large individuals ~ Level**			
**Analyzed group**	***p*-value**	**Adj. R^2**	**Spear. *p***	**Spear. ρ**	***p*-value**	**adj. R^2**	**Spear. *p***	**Spear. ρ**
Brachiopods + Bivalves	0.684	-0.018	0.707	-0.055	**<0.001**	0.334	**<0.001**	-0.560
Brachiopods	0.965	-0.022	0.696	0.058	**<0.001**	0.217	**0.001**	0.457
Bivalves	0.541	-0.013	0.665	0.064	**<0.001**	0.317	**<0.001**	-0.592

Table S1. Results of simple Ordinary Least Squares (OLS) and Spearman's Rank correlations of shell size and relative abundance of larger-sized individuals against time as represented by the sampling level. Statistically significant values are in bold. Abbreviations in table: Adj. R^2 = Adjusted R^2; Spear. *p*, Spear. ρ = *p*- and rho- values from the Spearman's Rank correlation test.

Table S2

		Shell Size ~ % Large individuals		
**Analyzed group**	**Lag**	**Autocorrelation**	**D-W Statistic**	***p*-value**
Brachiopods + Bivalves	1	0.403	1.124	**0.002**
	2	0.150	1.620	0.206
	3	0.058	1.589	0.232
	4	0.070	1.563	0.24
	5	0.269	1.122	**0.016**
Brachiopods	1	0.384	1.051	**0**
	2	0.155	1.429	0.066
	3	0.081	1.335	**0.044**
	4	0.081	1.333	0.060
	5	0.201	1.069	**0.012**
Bivalves	1	0.500	0.993	**0**
	2	0.377	1.217	**0.01**
	3	0.318	1.204	**0.01**
	4	0.250	1.323	**0.048**
	5	0.114	1.587	0.336

Table S2. Results of the Durbin-Watson test for autocorrelation of residuals at multiple lags of the Ordinary Least Squares (OLS) correlation of shell size and relative abundance of larger-sized individuals. Statistically significant results are marked in bold. Results given for the model before differencing.

Table S3

	X ~ Level				$\delta^{18}O$ ~ Level			
**Analyzed group**	***p*-value**	**Adj. R^2**	**Spear. *p***	**Spear. ρ**	***p*-value**	**adj. R^2**	**Spear. *p***	**Spear. ρ**
Body size (Brachiopods + Bivalves)								
Lag 0	0.892	-0.027	0.825	0.037	0.392	-0.007	0.201	-0.212
Lag 1	Results same as for lag 0*				0.381	-0.006	0.201	-0.215
Body size (Brachiopods)								
Lag 0	0.665	-0.023	0.556	0.100	0.271	0.007	0.154	-0.239
Lag 1	Results same as for lag 0*				0.257	0.009	0.156	-0.242
Body size (Bivalves)								
Lag 0	0.188	0.021	0.212	0.207	0.320	-0.007	0.201	-0.212
Lag 1	Results same as for lag 0*				0.381	-0.006	0.201	-0.215
% Large individuals (Brachiopods + Bivalves)								
Lag 0	**<0.001**	0.307	**<0.001**	-0.574	0.392	-0.007	0.201	-0.212
Lag 1	Results same as for lag 0*				0.560	-0.019	0.201	-0.215
% Large individuals (Brachiopods)								
Lag 0	**0.002**	0.225	**0.002**	-0.489	0.271	0.007	0.154	-0.239
Lag 1	Results same as for lag 0*				0.257	0.009	0.156	-0.242
% Large individuals (Bivalves)								
Lag 0	**<0.001**	0.253	**<0.001**	-0.597	0.387	-0.007	0.201	-0.215
Lag 1	Results same as for lag 0*				0.368	-0.005	0.201	-0.218

Table S3. Results of simple Ordinary Least Squares (OLS) and Spearman's Rank correlations of the faunal and isotope data against time as represented by the sampling level. Statistically significant values are in bold. Abbreviations in table: X = faunal data to be correlated; Adj. R^2 = Adjusted R^2; Spear. *p*, Spear. ρ = *p*- and rho- values from the Spearman's Rank correlation test. Results at lag 1 for the body size time series are the same as those at lag 0 because only the isotope time series was lagged.

Table S4

		X ~ δ^{18}O (lag 0)			X ~ δ^{18}O (lag 1)		
**Analyzed group**	**Lag**	**Autocorrelation**	**D-W Statistic**	***p*-value**	**Autocorrelation**	**D-W Statistic**	***p*-value**
Body size (Brachiopods + Bivalves)	1	0.269	1.400	**0.038**	0.255	1.464	0.068
	2	0.172	1.565	0.182	0.002	1.649	0.328
	3	0.061	1.517	0.246	0.053	1.536	0.272
	4	0.011	1.590	0.464	-0.093	1.786	0.842
	5	-0.119	1.827	0.894	-0.126	1.820	0.948
Body size (Brachiopods)	1	0.455	0.946	**0**	0.377	1.112	**0**
	2	0.182	1.379	0.082	-0.040	1.632	0.266
	3	-0.009	1.549	0.310	-0.012	1.533	0.280
	4	-0.045	1.602	0.502	0.024	1.401	0.180
	5	-0.083	1.610	0.648	-0.096	1.612	0.624
Body size (Bivalves)	1	0.182	1.596	0.166	0.147	1.682	0.292
	2	0.124	1.708	0.392	0.047	1.684	0.352
	3	0.226	1.233	**0.032**	0.156	1.440	0.164
	4	0.056	1.491	0.298	-0.104	1.865	0.918
	5	-0.128	1.858	0.838	-0.185	2.011	0.512
% Large individuals (Brachiopods + Bivalves)	1	0.422	1.106	**0.002**	0.209	1.532	0.116
	2	0.464	0.986	**0**	0.429	1.052	**0.006**
	3	0.351	1.160	**0.012**	0.326	1.190	**0.026**
	4	0.188	1.431	0.164	0.099	1.625	0.550
	5	0.174	1.395	0.256	0.155	1.337	0.166
% Large individuals (Brachiopods)	1	0.531	0.807	**0**	0.435	1.083	**0.002**
	2	0.303	1.213	**0.018**	0.253	1.302	**0.042**
	3	0.123	1.476	0.186	0.200	1.328	**0.056**
	4	0.116	1.470	0.276	0.086	1.525	0.348
	5	0.057	1.545	0.466	0.001	1.621	0.706
% Large individuals (Bivalves)	1	0.385	1.225	**0.004**	0.153	1.676	0.262
	2	0.361	1.250	**0.02**	0.409	1.157	**0.014**
	3	0.393	1.183	**0.014**	0.319	1.250	**0.056**
	4	0.353	1.084	**0.012**	0.221	1.258	**0.070**
	5	0.348	1.013	**0.012**	0.266	1.076	**0.032**

Table S4. Results of the Durbin-Watson test for autocorrelation of residuals at multiple lags of the Ordinary Least Squares (OLS) correlation of faunal and isotope data. “X” = faunal data correlated with δ^{18}O. Statistically significant results are marked in bold. The significant

autocorrelation at lag = 3 in bivalves is small enough that it can be ignored. The results for the percentage of larger-sized individuals for each of the three analyzed groups are given for the model before differencing.

REFERENCES CITED

1. Brand, U. *et al.* Oxygen isotopes and $MgCO_3$ in brachiopod calcite and a new paleotemperature equation. *Chem. Geol.* ***359***, 23–31, doi: 10.1016/j.chemgeo.2013.09.014 (2013).
